# Quantitative structure-activity relationship to elucidate human CYP2A6 inhibition by organosulfur compounds

**DOI:** 10.5599/admet.678

**Published:** 2019-08-05

**Authors:** Daniela A. Ramirez, Eduardo J. Marchevsky, Juan M. Luco, Alejandra B. Camargo

**Affiliations:** 1 Instituto de Biología Agrícola de Mendoza (IBAM), Consejo Nacional de Investigaciones Científicas y Técnicas (CONICET). Almirante Brown 500, Chacras de Coria, Mendoza, Argentina; 2 Área de Química Analítica, Facultad de Química, Bioquímica y Farmacia, Universidad Nacional de San Luis, Chacabuco y Pedernera 5700 San Luis, Argentina

**Keywords:** Quantitative structure-activity relationship (QSAR), CYP2A6, Inhibitors, Organosulfur compounds

## Abstract

CYP2A6 is a human enzyme responsible for the metabolic elimination of nicotine, and it is also involved in the activation of procarcinogenic nitrosamines, especially those present in tobacco smoke. Several investigations have reported that reducing this enzyme activity may contribute to anti-smoking therapy as well as reducing the risk of promutagens in the body. For these reasons, several authors investigate selective inhibitors molecules toward this enzyme. The aim of this study was to evaluate the interactions between a set of organosulfur compounds and the CYP2A6 enzyme by a quantitative structure-activity relationship (QSAR) analysis. The present work provides a better understanding of the mechanisms involved, with the final goal of providing information for the future design of CYP2A6 inhibitors based on dietary compounds. The reported activity data were modeled by means of multiple regression analysis (MLR) and partial least-squares (PLS) techniques. The results indicate that hydrophobic and steric factors govern the union, while electronic factors are strongly involved in the case of monosulfides.

## Introduction

Cytochrome P450s (CYPs) comprise a superfamily of heme-containing enzymes responsible for the metabolism of numerous compounds, therefore they are important objects of study mainly in the areas of pharmacology and toxicology [23]. CYPs are known for the diversity of reactions they catalyze, as well as, the range of different molecules with whom they interact. Cytochrome P450s are responsible for the majority of Phase I metabolism of exogenous molecules such as drugs, xenobiotics, environmental pollutants, dietary compounds, and endogenous molecules such as steroids, fatty acids, and prostaglandins [3,5,10]. The effects of these transformations can be manifested in drug bioavailability and toxicity, adverse drugs interactions, activation of procarcinogenic compounds, and biotransformation of molecules for subsequent elimination [8,9].

Among the CYP enzymes, CYP2A6 is the only member of the 2A subfamily that is expressed in humans and it catalyzes the coumarin 7-hydroxylation reaction (marker substrate)[5,6,23]. The current interest in CYP2A6, its polymorphisms and alleles, is mostly due to its effects on tobacco addiction, and the subsequent impact on lung cancer. Accordingly, two important features are noteworthy: CYP2A6 is the major catalyst of metabolic elimination of nicotine, and it is also involved in the activation of procarcinogenic nitrosamines, especially those present in tobacco smoke [14].

On the other side, nicotine is the essential component that causes tobacco dependence. The pharmacological, as well as, physiological effects of smoking are due to this alkaloid. Pulmonary absorption of nicotine is extremely rapid, and once it is absorbed, it is rapidly and extensively metabolized and eliminated through urine. In general, 70-80 % of its biotransformation consists mainly in the oxidation of (-)-nicotine to form (-)-cotinine mediated by CYP2A6. Nicotine is also metabolized to nornicotine, via *N*-demethylation. In humans, 2-3 % of nicotine is excreted as nornicotine in 24 h urine [5,20,21]. Several nicotine preparations have been developed as a medication to assist in smoking cessation; also other therapies and treatments have been proposed to reduce tobacco consumption, among them, we can name the use of nicotine patches, dopamine uptake inhibitor (bupropion), consumption of tricyclic antidepressants and buspirone anxiolytic. However, the previous therapies are of limited use because of the secondary effects that come with them and due to a low proven success (below 40%), for this reasons alternative treatments are encouraged [22]. With this goal, several investigations have shown that the inhibition of CYP2A6 by different chemical compounds may represent a potential supplement to anti-smoking therapy [12]. The efficiency of CYP2A6-mediated biotransformation of nicotine is associated with nicotine blood levels for keeping the addiction liability. Potent and specific inhibitors of CYP2A6 might increase nicotine half-life elimination time. Consequently, the inhibition of CYP2A6 results in a diminished desire to smoke and a reduced consumption of toxic or carcinogenic components of cigarette smoke [17]. Moreover, the administration of chemicals that strongly and specifically inhibit the CYP2A6 activity might also result in a reduction of mutagens in the body, since the N-nitrosamines activation does not occur when this enzyme is altered [5].

Numerous compounds have been proposed and tested as CYP2A6 inhibitors, among them methoxsalen, (R)-(+)-menthofuran, and tranylcypromine can be mentioned. Even though they have shown strong inhibitory effects, methoxsalen and tranylcypromine also inhibited other forms of CYP [5]. On the other hand, *in silico* studies, such as QSAR models, serve to predict and explain molecular properties and/or biological activities from the structure of chemical compounds reducing the number of tests, thereby resulting in a quick and simple alternative for designing new drugs with a desirable effect. In this sense, some authors have carried out *in silico* studies to predict selective CYP2A6 inhibitors based on a series of nicotine derivatives [17], naphthalene and non-naphthalene derivatives [11,15], or based on virtual screening of a large number of compounds [13]. To date, all the studied *in silico* inhibitors are from synthetic origin. However, it has been proved that some sulfide and disulfide compounds present in *Allium* vegetables, inhibit CYP activity. In this sense, the anticarcinogenic and antitumorigenic effects of garlic in rodents have been attributed to modulation of CYP activity for some OSCs such as diallyl sulfide (DAS), diallyl disulfide (DADS) and allyl methyl sulfide (AMS) [7,16]. Fujita and Kamataki, (2001) [5] studied the inhibition of CYP2A6 by determining the ability of 22 organosulfur compounds to inhibit coumarin 7-hydroxylase activity. Based on these experimental values, we carried out a quantitative structure-activity relationship (QSAR) analysis to evaluate these inhibitors-enzyme interactions in order to obtain a wider vision into the mechanisms involved. In turn, a potential selective inhibitor of CYP2A6 based on naturally occurring compounds can be proposed. The reported activity data were modeled by means of multiple regression analysis (MLR) and partial least-squares (PLS) techniques. For the quantitative description of the analyzed structures, different quantum chemical indices (AM1) and physicochemical parameters were calculated. Non-empirical descriptors, such as topological and geometrical, were also used as descriptive variables. Finally, the obtained results provided an explanation about which are the main features and parameters of OSCs (lipophilicity, steric and electronic features) that contribute to an optimal inhibitory activity on CYP2A6.

## Materials and methods

### Biological and chemical data

The chemical structures along with observed activity data of the compounds used in this study are shown in Table 1. The inhibitory activity data on CYP2A6 were obtained from Fujita and Kamataki, (2001) [5], and it was expressed as percentage of Residual Activity (RA%) when using 10 μM of each inhibitor and 2.5 μM of coumarin. In order to evaluate whether the biological data comes from a normal distribution, the standardized skewness and kurtosis values were calculated for RA% (n=22).

### Structural descriptors

A set of molecular descriptors were calculated to characterize the compounds under study. As for indicators of molecular size, the following were considered: molar volume (Vm), molecular weight (MW), and molar refractivity (MR). To explain lipophilicity effects, several calculated partition coefficients were obtained by using ALOGPS 2.1 software (log *P*_exp_, ALOGPs, IA Log *P*, COSMOFrag, miLog *P*, KOWWIN and XLOGP). Another group of structural descriptors included quantum chemical indices obtained by HyperChem package (release 7.5 for Windows). Three-dimensional molecular structures were built using the MM+ molecular mechanics potential-energy function. In a follow-up procedure, complete optimization of the geometrical parameters was carried out by using the AM1 method, implemented in the standard version of MOPAC 6.0. The following indices obtained from molecular orbital calculations were considered: total energy (*E*_total_), heat of formation (¢H_f_), energy of highest occupied molecular orbital (HOMO), energy of lowest unoccupied molecular orbital (LUMO), dipole moment (*μ*), absolute total charge (*Q*_total_), the most positive and the most negative absolute charges (*q*_p,max_, *q*_n,max_), and the positive and negative relative charge (RNCG, RPCG). The last group of descriptors considered in this study included various geometrical and topological indices: the Wiener index, the valence and connectivity molecular indices, the kappa shape indices, and several geometrical indices calculated from the optimized distance matrix AM1 by using Dragon software (v 3.0). A stepwise multiple regression procedure, based on the algorithms *forward-selection* and *backward-elimination*, was used for the inclusion or rejection of descriptors in the screened models.

### Statistical methods

Partial least squares projections in latent variables (PLS) together with multiple regression analysis (MLR) were the selected methods to search for relationships between the biological activity data and the structural descriptors. The determination of the significant number of PLS components was made by crossvalidation [1,19]. PLS analysis was carried out using the SIMCA-P 7.01 software obtained from Umetri AB, Umea, Sweden, and MLR analysis was performed using the 7.0 version of Statgraphics Plus software. Prior conducting the PLS-1 analysis for each one of the classes, all the variables were auto-scaled to zero mean and unit variance. This transformation assures data standardization and allows to give each descriptor equal importance in the PLS analysis. To avoid overestimations or misunderstanding interpretation of the resulting models, pairs of variables with r ≥ 0.75 were classified as intercorrelating ones, and only one of these was included in the screened model. The predictive ability of the model was evaluated by the crossvalidation coefficient (Q^2^), which is based on the prediction error sum of squares (PRESS). The PRESS statistic is computed as the squared differences between observed and predicted values when the observations are kept out of the derived model. This procedure is repeated several times until every observation has been kept out once, and only once.

## Results

### Multiple regression analysis

MLR was performed on the 22 OSCs described in Table 1, whereas Table 2 shows the molecular descriptors included in the selected models.

First, in order to evaluate the data set distribution the standardized skewness and kurtosis values were calculated for RA% (n=22). Skewness is a measure of symmetry, or more precisely, the lack of symmetry; and Kurtosis indicates whether the data distribution is heavy-tailed or light-tailed relative to a normal distribution. In other words, data sets with high kurtosis tend to have heavy tails, or outliers. The obtained results considering RA% were -1.5517 and -0.2634 for the standardized skewness and kurtosis values, respectively. Values outside -2 to +2 range indicate significant departures from normality. In this case, the obtained values are within the expected range for data presenting normal distribution.

On the other hand, because of the large number of structural descriptors considered in this study, the VIP (variable importance for the projection) parameter [18] was used to unravel which descriptors were relevant to explain the enzyme activity. VIP is based on the PLS projection method, which constitutes a robust method for variables selection.

Considering the arguments presented by Yano et al. (2006) [22] along with other background QSAR studies about CYP2A6 enzyme, which showed that the nature of the enzyme active site is strongly hydrophobic, it was decided first, to study the relationship between activity and lipophilicity. After using several calculated log *P* parameters, the obtained equations showed no significant correlations in this direction. On the other hand, since lipophilicity can be described by the contribution of the molecular size and molecular polarity, models that incorporated both molecular properties were investigated. The best regression equation that includes the parameter CLOGP, was:


(1)

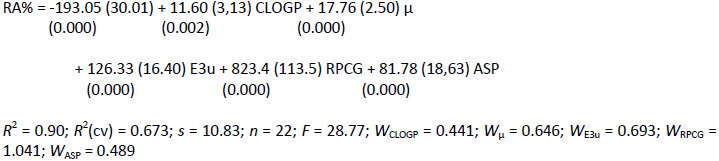



In equation (1) and the following equations, *n* is the number of compounds, *s* is the standard deviation, *R*^2^ is the squared correlation coefficient, *R*^2^(cv) is the squared crossvalidation coefficient, and *F* is the Fisher *F* statistic. Values in parentheses correspond to the standard deviations and *p-*values of coefficients, and the term *W*, represents the standardized regression coefficient when variables are scaled to the same numerical range (0-1).

The statistical quality of the model is suitable. Although the relationship between numbers of observations versus the number of variables is high, the good quality in fit and predictive ability, as expressed by *R*^2^ and *R*^2^(cv) coefficients, suggests that the obtained model is valid.

Other models were also investigated, in which the relationship between observations / variables were lower in relation to the previously derived equation.


(2)

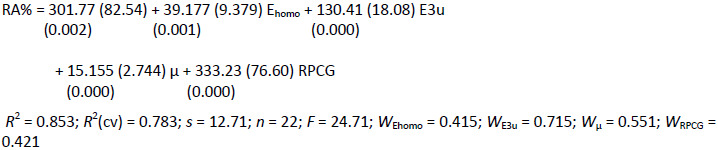



**Figure 1.**
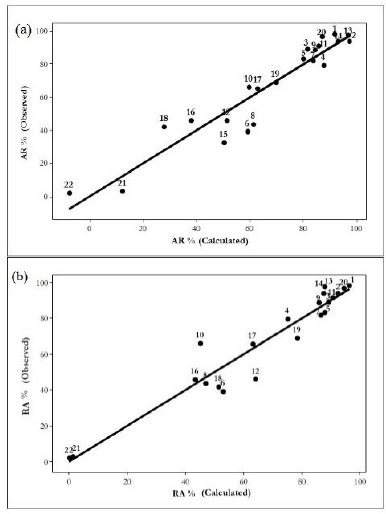
Relationship between observed and calculated values for **(a)** equations (1) and **(b)** equation (2)

This equation is highly significant, and the molecular descriptors here used, did not show significant intercorrelations. Figure 1 shows the relationships between the experimental and calculated Residual Activity (RA%) for equations (1) and (2), respectively. An interesting point to highlight in this equation is an improvement in the crossvalidation coefficient value, which suggests a better predictive ability.

An alternative way to evaluate the reliability and robustness of a QSAR model consists in applying different statistical approaches, but using the same descriptors that have been included in the original model. Accordingly, a linear discriminant analysis (LDA) was carried out based on the molecular descriptors used in equation (2) to maintain an adequate balance between the numbers of observations vs. the number of independent variables. Taking into account the information from the inhibitory activity data, the compounds were separated into three groups, namely highly active (RA% 1-10), active and weakly active (RA% 11-70) and weakly active or inactive (RA% 71-100). The discriminant ability was assessed by the correct classifications percentage, and the discriminant function quality was evaluated using the Wilks parameter, λ, which was obtained by a multivariate analysis of variance that tests the equality of group means for the variable in the discriminant model. The first standardized discriminating function was:


(3)

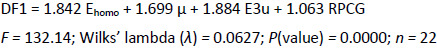



**Figure 2.**
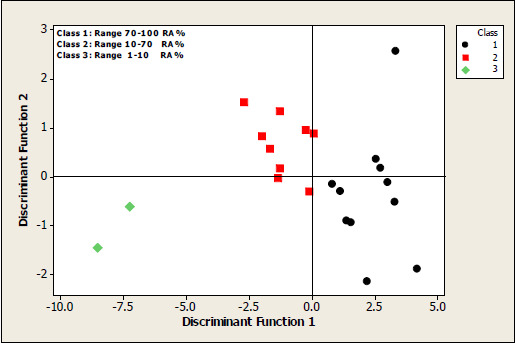
2-D plot of discriminant functions DF1 and DF2.

This equation is highly significant as P < 0.00001, and amongst the 22 observations used to fit the model, 22 (or 100.0 %) were correctly classified. In order to check an overestimation of the obtained model, the rate of correct classification was evaluated by using crossvalidation and a 95.5% was obtained (21 to 22). From the relative magnitude of the obtained coefficients in equation (3), it can be appreciated the contribution of a single variable to discriminate amongst the groups. Figure 2 plots each observation in the space of the two linear discriminant functions (DF1, DF2).

The fact that similar conclusions are obtained by using both kinds of statistical methods; that is, MLR and LDA, provides more evidence about the model robustness here reported.

The data set, includes both monosulfides as disulfides. In the case of monosulfides, these can act as strong nucleophiles (Nu^θ^) or reducing agents depending on the nature of the electrophile(E^⊕^), the substituent group (R-S), and the medium in which the reaction occurs. Such property is related to the E_homo_ energy of monosulfides, thus, a greater E_homo_ implies a higher ionization potential and a superior ability to donate electrons. Moreover, disulfides act as electrophilic oxidants, i.e. tend to accept electrons, which is reflected by their low LUMO energy orbital. Considering the above, the set of compounds under study was divided among mono- and disulfides, which were analyzed independently. The relationship between monosulfides and their corresponding E_homo_ energy showed a parabolic correlation as presented in Equation (4).


(4)

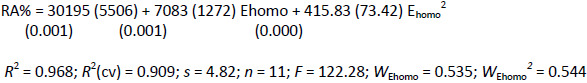



The quadratic term E_homo_ is statistically significant (p = 0.000), although this term depends very strongly on compound **16**. Thus, only the analysis of a data serie including a larger amount of monosulfides would help to validate the parabolic relationship obtained. Another aspect to highlight, is that compound **21** (4,4'- dipyridyl sulfide) was excluded from the analysis because it presented an *outlier* behavior, given its polarity and structural nature, that differs from the other monosulfides (this may be due to the fact that compound ***21*** is the only pyridyl monosulfide compound).

In the case of the 10 disulfides under study, the best-obtained regression equation was:


(5)

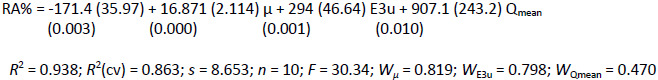



**Figure 3.**
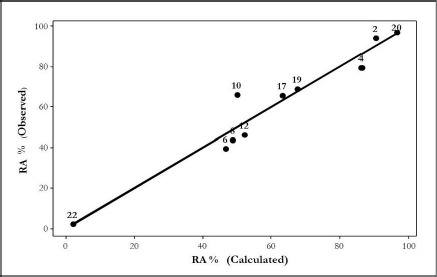
Relationship between observed and calculated values for equation (4).

In Figure 3, it is possible to observe an agreement between the experimental and calculated values of RA%, as well as the normal distribution of residual values. The values of the correlation coefficient (*r*) between pairs of variables used in this equation showed an acceptable intercorrelation: μ/E3u -0.099, μ/Q_mean_ -0.028, and E3u/Q_mean_ -0.586.

### Partial least square-1 regression analysis

A preliminary analysis of different PLS models performed on the whole series of compounds showed that the most important variables were those that had been useful in the regression analysis. Finally, the analysis of molecular descriptors was performed using PLS-pseudoregression coefficients, and the statistical significance of all QSAR-PLS derivatives models was evaluated by means of the following statistical parameters: variance of the matrices X and Y (R2X and R2Y), the correlation coefficient (r), standard deviation (RMSS) and the statistical F.

The PLS model was built using 21 of the total set of OSCs; compound **15** (allyl phenyl sulfide) was not included in the model because of its outlier behavior, as observed in MLR-QSAR models. The PLS-1 analysis resulted in two statistically significant components (CPLS), which statistical parameters are detailed in Table 3.

**Figure 4.**
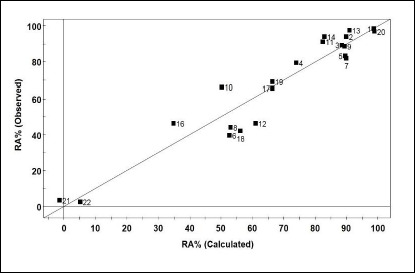
Relationship between observed and calculated values by the PLS-1 model derived.

**Figure 5.**
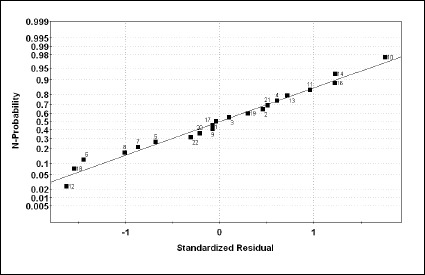
N-probability plot of residuals from the PLS-1 model.

**Figure 6.**
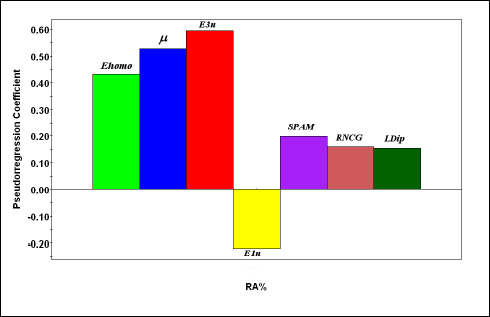
Bar graph of the standardized coefficients of the PLS-1 model.

Figure 4, shows the relationship between RA% experimental values and the corresponding calculated values derived from the PLS-1 models. As can be observed in Figure 5, the normal probability plot of the residuals is approximately linear, which indicates that the error terms are normally distributed. Moreover, the corresponding pseudoregression coefficients are shown in Figure 6. From these values, it can be observed how much a single variable contributes to RA% modelling.

### Validation of PLS-1 model

The actual predictive ability of a QSAR model can be judged using compounds not included originally in the calibration series, or alternatively, by using a permutation method [4]. Thus, to show that the developed PLS-1 model was not a result of chance, both validation methodologies were used. RA values [%], predicted for 4 unused compounds are shown in Table 4. As can be seen, predictions are within the range of experimental error, so they are quite acceptable. It should be mentioned that the classification model using LDA, as previously shown in equation (3), correctly predicts the activity of the four inhibitors shown in Table 4.

**Figure 7.**
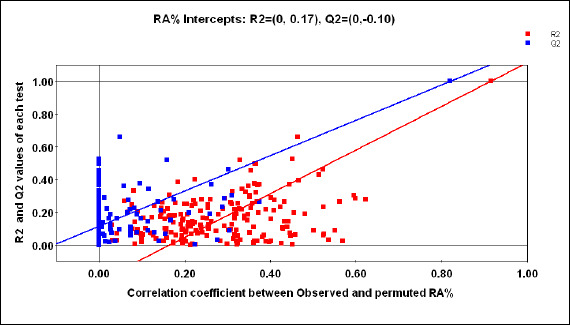
Permutation graph of the PLS-1 derived model.

As previously mentioned, the validity of the PLS-1 model was additionally tested by a permutation test. Models were recalculated for randomly reordered response data (RA%). These permuted RA% values were related to intact predictor data by refitting the model and including crossvalidation. When *R*2 and *Q*2 were plotted as a function of the correlation coefficient between the original values and the predicted values, the interception point with the *Y* axis expressed how much these values rely on chance. In Figure 7, the corresponding plot of RA% response permutation test is presented. The vertical axis gives the R2 and Q2 values of each test. The horizontal axis represents the correlation coefficient between the observed and the permuted RA% values (200 permutations of the RA% dependent variable under study).

The intersection of the two regression lines (for R^2^ and *Q* ^2^) in the figure indicates the degree of overshooting and predictability. In general, the valid model limits include values of R^2^ <0.30 and *Q* ^2^ <0.30. This condition is fulfilled, indicating that the PLS-1 model obtained is suitable both, for its adjustability as for its predictive ability.

## Discussion

The QSAR models here proposed showed the main factors prevailing the OSC-CYP2A6 binding. Several statistical analyses were carried out for this purpose. Four equations resulted from the Multiple Regression Analysis. The presence of CLOGP in equation (1) suggests that the molecular lipophilicity plays an essential role in the inhibition of CYP2A6. This fact was experimentally demonstrated by Yano et al. (2006) [22] and corresponds with the hydrophobic nature of the active site. On the other hand, equation (1) shows a high influence of the molecular shape in the inhibition process, as expressed by the ASP and E3u descriptors. The presence of the ASP (asphericity) descriptor, which is a measure of the spherical shape deviation of the molecule, allows us to infer that at higher molecular elongation, a more significant inhibitory activity can be observed. Regarding the E3u geometric descriptor, which belongs to the directional WHIM descriptors encoding the planar characteristics of the molecules, it will acquire values tending to zero for molecules ideally planar, and values tending to one for molecules that deviate from this property. Thus, considering equation (1) for the compounds under study, it was observed that compounds having lower E3u descriptor value, generally exhibit a higher inhibitory capacity.

Furthermore, from the analysis of equation (1), it was observed that the electronic factors are also involved in the inhibition of CYP2A6, as expressed by μ and RPCG descriptors. Hence, these descriptors reflect the ability of the compounds to participate in polar interactions, suggesting that higher molecular polarity will correspond to lower activity.

An interesting feature to consider from Equations (2) and (3) is the highly significant contribution of the electronic parameter E_homo_. The cavity in the substrate-binding site of CYP2A6 is strongly hydrophobic and this characteristic is due to the presence of several amino acid residues of phenylalanine. Thus, E_homo_ parameter suggests a π-π and / or n-π interaction between OSCs and phenylalanine residues.

According to previous studies concerning the catalytic versatility of cytochrome P450 [2], regarding the functional active oxygen of heme groups, it has been postulated that this group shows nucleophilic and/or electrophilic properties catalyzing different type of reactions such as aldehyde deformilations, epoxidations and/or hydroxylation.

On another note, the analysis of equation (4), which only considers monosulfides, suggests that the electrons donor ability of these compounds, expressed through E_homo_ parameter, plays a critical role in the interaction with CYP2A6. Thus, at higher E_homo_ energy of monosulfides, a greater inhibitory capacity can be observed. In short, from the analysis of the equation, it is possible to infer that the monosulfides could act as nucleophiles and interact with hydroperoxo-iron forms and/or oxenoid-iron from Heme group. However, the interaction efficiency between monosulfides-CYP2A6 also depends on the hydrophobicity, or alternatively on the lower polarity that these compounds present, as reflected by Equations (1) and (2).

An important aspect to mention is that the E_Lumo_ parameter was not significant as a descriptor variable in any of the models investigated. This fact suggests that in the case of the disulfides, the ability to accept electrons was not relevant to the CYP2A6-disulfide interaction. Thus, it is possible to postulate that the inhibitory potency of the disulfides depends mainly on geometric and electronic factors.

To further investigate the OSCs-CYP2A6 binding, a PLS-1 analysis was carried out. Analyzing the model coefficients (Figure 6), it was observed that the geometric terms E1u, E3u, and SPAM play an essential role in the model fit, in agreement with the MLR technique results. This aspect indicates the crucial role of steric factors (including non-specific Van der Waals interactions) in the inhibitory activity exerted by such compounds. The other molecular parameters showed the importance of the electronic aspects of the OSC-CYP2A6 interaction. The presence of RNCG, *μ* and L*Dip* coefficients in the model indicates that an increase in the molecular polarity plays a negative effect on the inhibitory activity of the OSCs; which fully corresponds with the current knowledge on the hydrophobic nature of the active site. Finally, the model showed that in the case of monosulfides, E_homo_ electronic parameter, or the ability to donate electrons, is a significant factor in the enzyme-inhibitor binding.

## Conclusions

This work allows obtaining a better understanding of the mechanisms involved between the enzyme CYP2A6 and OSCs. The obtained results indicate that hydrophobic and steric factors govern the union, while electronic factors via the electrons donor ability are strongly involved in the case of monosulfides, which could act as nucleophiles and interact with hydroperoxo-iron forms and/or iron-oxenoid from Heme groups. It was also possible to conclude that the inhibitory effect of the disulfides depends mainly on geometric and electronic factors. Furthermore, it was determined that an increase in the molecular polarity exerts a negative effect on the inhibitory activity of the tested compounds, which corresponds with previous studies that indicate a high hydrophobicity of the enzyme active site.

On the other side, this study provides evidence of the enormous potential of WHIM descriptors for the development of QSAR models.

## Figures and Tables

**Table 1.** CYP2A6 Inhibitory Activity Data of Organosulfur Compounds

N°	Name	Chemical structure	Residual Activity %
1	Dimethyl sulfide	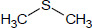	98.4
2	Dimethyl disulfide	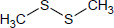	94.1
3	Diethyl sulfide	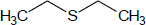	89.4
4	Diethyl disulfide	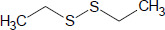	79.6
5	Di-n-propyl sulfide	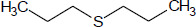	83.4
6	Di-n-propyl disulfide	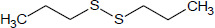	39.5
7	Di-n-butyl sulfide	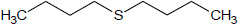	82.0
8	Di-n-butyl disulfide	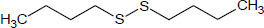	43.9
9	Di-n-amyl sulfide	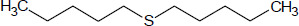	88.8
10	Di-n-amyl disulfide	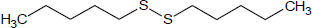	66.3
11	Diallyl sulfide	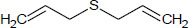	91.3
12	Diallyl disulfide	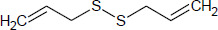	46.2
13	Allyl methyl sulfide	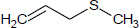	97.6
14	Allyl n-propyl sulfide	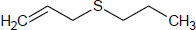	94.2
15	Allyl phenyl sulfide	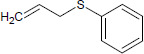	32.8
16	Diphenyl sulfide	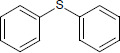	46.1
17	Diphenyl disulfide	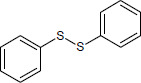	65.7
18	Phenyl ciclopropyl sulfide	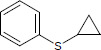	42.1
19	Difurfuryl disulfide	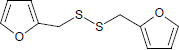	69.1
20	4,4’-dipyridyl disulfide	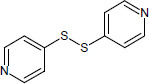	97.0
21	4,4’-dipyridyl sulfide	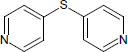	3.22
22	2,2’-dipyridyl disulfide	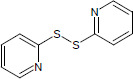	2.26

**Table 2.** Molecular Descriptors^a^ for the compounds described in Table 1

N°	RA%	CLOGP	E_homo_	E_lumo_	μ	LDip	RPCG	RNCG	E1u	E3u	SPAM	ASP	*Q*_mean_
1	98.400	0.845	-8.882	0.457	1.961	0.251	0.200	0.484	0.707	0.444	0.517	0.237	0.093
2	94.100	0.845	-9.399	-1.978	2.569	0.249	0.193	0.473	0.724	0.449	0.545	0.235	0.095
3	89.400	1.817	-8.856	0.362	1.941	0.154	0.118	0.282	0.654	0.533	0.488	0.665	0.076
4	79.600	1.817	-9.352	-1.976	2.646	0.160	0.132	0.289	0.676	0.484	0.504	0.521	0.078
5	83.400	2.604	-8.857	0.353	1.910	0.146	0.090	0.216	0.632	0.571	0.480	0.765	0.074
6	39.500	2.604	-9.351	-1.996	2.660	0.151	0.103	0.230	0.677	0.358	0.489	0.547	0.075
7	82.000	3.291	-8.861	0.342	1.886	0.134	0.071	0.170	0.612	0.593	0.475	0.875	0.073
8	43.900	3.291	-9.351	-1.999	2.676	0.139	0.083	0.183	0.642	0.367	0.477	0.659	0.074
9	88.800	3.911	-8.865	0.337	1.881	0.129	0.059	0.141	0.605	0.606	0.472	0.900	0.072
10	66.300	3.911	-9.354	-2.002	2.677	0.133	0.069	0.153	0.643	0.374	0.473	0.681	0.073
11	91.300	2.352	-8.901	-0.068	2.004	0.153	0.133	0.169	0.594	0.348	0.525	0.763	0.100
12	46.200	2.352	-9.313	-2.010	2.650	0.157	0.127	0.171	0.626	0.294	0.515	0.346	0.102
13	97.600	1.672	-8.894	0.146	1.987	0.187	0.178	0.314	0.590	0.310	0.527	0.604	0.098
14	94.200	2.459	-8.880	0.108	1.958	0.150	0.139	0.209	0.594	0.353	0.518	0.778	0.086
15	32.800	3.437	-8.566	-0.076	1.649	0.140	0.113	0.161	0.574	0.227	0.514	0.642	0.105
16	46.100	4.412	-8.371	-0.142	1.265	0.127	0.130	0.127	0.613	0.000	0.474	0.615	0.110
17	65.700	4.412	-8.960	-2.164	2.495	0.139	0.096	0.188	0.624	0.331	0.477	0.484	0.105
18	42.100	3.527	-8.557	0.086	1.627	0.158	0.105	0.217	0.618	0.172	0.467	0.524	0.112
19	69.100	1.182	-9.143	-2.170	2.191	0.126	0.123	0.152	0.627	0.376	0.519	0.749	0.101
20	97.000	1.406	-9.320	-2.365	5.911	0.140	0.108	0.158	0.602	0.248	0.498	0.471	0.105
21	3.220	1.406	-9.007	-0.667	0.321	0.135	0.160	0.133	0.620	0.000	0.487	0.630	0.113
22	2.260	1.406	-9.573	-2.627	0.150	0.121	0.111	0.160	0.602	0.248	0.500	0.534	0.108

^a^ For an explanation of the symbols, refer to the text

**Table 3:** Statistical parameters and PLS model

Comp.	R2X(CUM)	R2Y(CUM)	Q^2^(CUM)	RMSS	F statistical
CPLS-1	0.400	0.587	0.418	9.1347	122.380
CPLS-2	0.566	0.914	0.817		
(r=0.965) (q=0.904)					

**Table 4.** Predictions from the derived QSAR model

Known inhibitors	Activity (observed)^a^	Activity (predicted)^b^	PModX (PS)^c^
Nicotine	98.1	91.614	0.03391
Tranylcypromine	12.9	29.952	0.31252
Cotinine	96.2	99.324	0.00343
SM-12502	85.9	84.476	0.01204

^**a**^ Activities expressed as RA% (Source: Fujita and Kamataki, 2001)

^**b**^ Predicted activity values (RA%) using the developed PLS-1 model

^**c**^ Probability of belonging to the descriptor space used in the model. In this case, tranylcypromine and SM-12502 they belong, while nicotine and cotinine are borderline.
